# Screening of SNP Loci Related to Leg Length Trait in Leizhou Goats Based on Whole-Genome Resequencing

**DOI:** 10.3390/ijms252212450

**Published:** 2024-11-20

**Authors:** Jinyang Liu, Shucan Dong, Jianda Lv, Yaokun Li, Baoli Sun, Yongqing Guo, Ming Deng, Dewu Liu, Guangbin Liu

**Affiliations:** College of Animal Science, South China Agricultural University, Guangzhou 510642, China; liujinyang@stu.scau.edu.cn (J.L.); dsc15184989294@163.com (S.D.); 18633565621@163.com (J.L.); ykli@scau.edu.cn (Y.L.); baolisun@scau.edu.cn (B.S.); yongqing@scau.edu.cn (Y.G.); dengming@scau.edu.cn (M.D.)

**Keywords:** Leizhou goat, leg length trait, whole-genome resequencing, SNPs

## Abstract

Leizhou goats can be classified into tall and short types based on their size and habits. The tall Leizhou goats are well-suited for grazing management due to their robust physique, while the dwarf types are smaller, grow rapidly, and are more appropriate for feeding management systems. In this study, whole-genome resequencing was conducted to identify genomic variants in 15 Tall-legged (TL) and 15 Short-legged (SL) Leizhou goats, yielding 8,641,229 high-quality SNPs in the Leizhou goat genome. Phylogenetic tree and principal component analyses revealed obvious genetic differentiation between the two groups. Fst and θπ analyses identified 420 genes in the TL group and 804 genes in the SL group. Gene Ontology (GO) and Kyoto Encyclopedia of Genes and Genomes (KEGG) pathway analyses indicated that the phosphatidylinositol signaling system is associated with growth and development. Additionally, Genome-Wide Association Study (GWAS) analysis identified eight genes linked to leg length, including *B4GALT7* and *NR1D1*. Notably, the NC_030818.1 (g.53666634T > C) variant was significantly associated with leg length traits, where the CC genotype was linked to shorter legs and the TT genotype to longer legs. This study identifies candidate genes and molecular markers, serving as a reference point for breeding and genetic improvement efforts in Leizhou goats and other goat breeds.

## 1. Introduction

**Figure 1:**
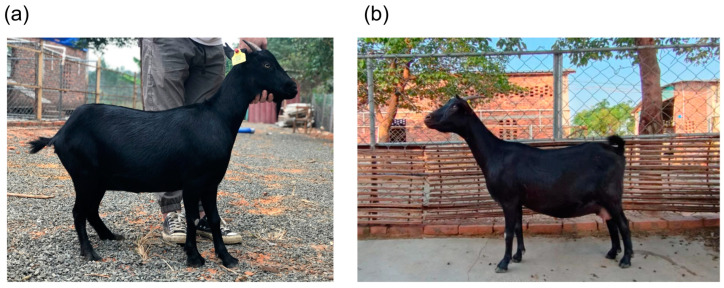
Short-legged (SL) Leizhou goats: (**a**); tall-legged (TL) Leizhou goats: (**b**).

Leizhou goats are native to the Leizhou Peninsula in Guangdong Province, China, and exhibit notable advantages such as strong disease resistance, superior meat quality, rapid growth, and high reproductive rates [1]. Leizhou goats can be categorized into tall-legged and short-legged types based on leg morphology (Figure 1). Compared to the short-legged type, the tall-legged goats have a larger body, smaller abdomen, and less developed udder, resulting in a higher proportion of single-kidding females, making them more suitable for grazing. Conversely, the short-legged type has a smaller body, finer skeletal structure, larger abdomen, and a more developed udder. Although less mobile, they grow faster, have a higher meat yield, and exhibit superior reproductive performance, with a higher incidence of twin births, making them ideal for intensive farming. Identifying and selecting genes related to leg length is crucial for developing optimized indoor-raised Leizhou goat breeds.

Whole-genome resequencing (WGS) has become an important method for exploring breed-specific and selection traits in livestock [2]. WGS can comprehensively and accurately identify SNP variations through high-throughput sequencing of the entire genome. This comprehensively reveals genetic variation in the goat genome and has been used in selection and breeding programs. Traits such as high reproductive performance, good palatability, and high cashmere quality can be directly selected through the published genome maps. Extensive research has identified numerous candidate genes associated with quality traits in goats. Studies by Wang et al. [3] and Xiong et al. [4] have uncovered genes linked to wool and milk yield traits, reproduction, and immunity, such as *SEMA3D*, *EVPL*, *FGF12*, *SOX5*, *DGAT2*, *GHR*, *ELF5*, and *GLYCAM1*. Gao et al. [5] expanded this list through a resequencing study, highlighting genes like *C2CD3*, *UCP2*, and *TSHR* that influence nervous system functions, growth, and coat color. Lan et al. [6] further contributed to this body of work by identifying the *POU1F1 gene* variant associated with litter size, milk yield, and weight gain in goats. Collectively, these findings underscore the genetic diversity that contributes to the phenotypic variation observed across different goat breeds. These studies underscore the potential of whole-genome resequencing in identifying breed-specific variation and supporting the development of new breeds.

Currently, research on growth traits in Leizhou goats remains limited. Existing studies predominantly focus on meat quality and lambing rates in reproductive traits and their associations with genetic polymorphisms. Wang et al. [7] found that the *plectin (PLEC) gene* has a regulatory role in muscle development in Leizhou goats and subsequently identified three copy number variants in the *PLEC gene* in 417 Leizhou goats. PLECCNV-1 was significantly associated with body weight, chest circumference, carcass weight, cross-sectional area of the longissimus dorsi muscle of the back, and shear force in Leizhou goats, and the type of weight gain was highly significantly correlated with the expression of *PLEC* in muscle. Mutations in POU1F1, a positive regulator of growth hormone (GH), prolactin (PRL), and thyroid-stimulating hormone β-subunit (TSHβ) in ruminants, were associated with growth traits. At present, the number of studies on the traits of Leizhou goats is still relatively small, and the use of modern molecular technology to screen for growth trait-related genes of Leizhou goats can provide a reference for the subsequent selection and breeding.

## 2. Results

### 2.1. Whole-Genome Resequencing and Mutation Detection in Leizhou Goats

#### 2.1.1. Quality Control and Comparison of Whole-Genome Resequencing Data

A total of 855.972 GB of raw sequencing data was generated from 30 Leizhou goat samples, yielding 851.26 GB of clean data after quality control. The results of sequencing statistics are presented in Table 1. The valid data rate for the 30 samples ranged from 98.11% to 99.45%, the Q30 values ranged from 91.24% to 93.34%, and the GC content ranged from 41.73% to 47.05%, with no evidence of significant GC bias. These outcomes confirm the reliability of the Illumina sequencing library construction and data quality. Alignment of the high-quality sequencing data to the reference genome (https://ftp.ncbi.nlm.nih.gov/genomes/all/GCF/001/704/415/GCF_001704415.1_ARS1/, accessed on 19 July 2022) was performed using BWA (0.7.8) software. On average, 99.67% of the assembled sequences were successfully mapped to the reference genome, with an average sequencing depth of 7.9 × per sample. The proportion of bases with a depth greater than once covered 94.39% of the reference genome, and the coverage at four times was 83.96% (Table 2). These results indicate that the similarity between the samples and the reference genome meets the requirements for resequencing analysis, providing sufficient depth and coverage for downstream analyses.

#### 2.1.2. SNP Variant Detection and Annotation

The distribution of these SNPs across the genome is detailed in Table 3. A total of 20,351,884 original SNP loci were identified, with 8,641,229 high-quality SNP loci retained after quality control screening. SNPs were annotated using ANNOVAR (21 Jun 2013) software, revealing that 5,455,335 SNPs (63.13%) were located in intergenic regions, 2,976,338 SNPs (34.44%) in intronic regions, 120 SNPs (0.0014%) in splice sites, 47,182 SNPs (0.55%) in regions 1 kb downstream of genes, and 1030 SNPs (0.012%) in regions 1 kb upstream. Additionally, 59,878 SNPs (0.69%) were located in exonic regions, with 35,791 (0.41%) being synonymous mutations, 23,807 (0.28%) non-synonymous mutations, and 280 (0.0032%) able to change the termination codon of the gene.

### 2.2. Population Structure Analysis of Leizhou Goats

**Figure 2:**
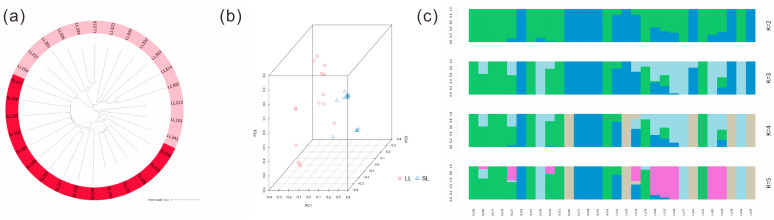
Population structure analysis of Leizhou goats: (**a**) phylogenetic tree of 30 Leizhou goats; (**b**) PCA diagram of principal component analysis of 30 Leizhou goats; (**c**) structure of 30 Leizhou goats.

In this study, Treebest was used to construct an evolutionary tree for 30 Leizhou goats, and the results are shown in Figure 2a. Most of the TL Leizhou goat groups were clearly genetically differentiated from the SL Leizhou goat groups, but three TL goats were slightly mixed into the SL goat groups. The results of principal component analysis (PCA) showed that the 30 Leizhou goats were able to be divided into two groups (Figure 2b). Structure analysis confirmed a clear separation between the two groups at K = 2 (Figure 2c)

### 2.3. Signatures of Selection in Leizhou Goat Populations

#### 2.3.1. Leizhou Goat TL vs. SL Based on Fst

**Figure 3:**
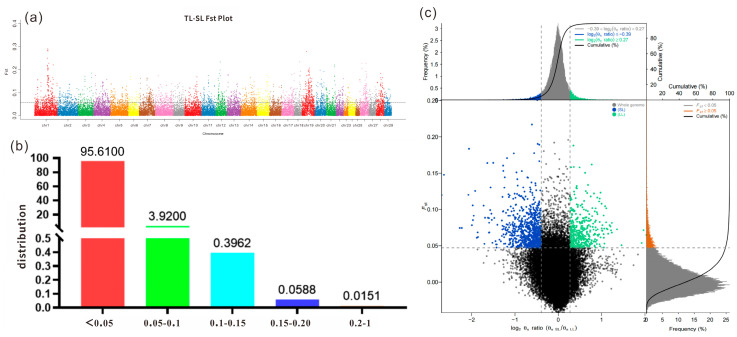
Selection elimination analysis of Leizhou goat populations: (**a**) autosomal Fst distribution of TL vs. SL group in Leizhou goat; (**b**) Fst ratio of Leizhou goat TL vs. SL group; (**c**) Leizhou goat TL vs. SL group Fst and θπ selection elimination analysis plot (The gray dashed line represents the threshold line for selection signals).

Fst selection analysis was conducted to identify candidate genes related to leg length traits by comparing genetic differences between TL and SL Leizhou goats (Figure 3a). The results showed that 95.61% of Fst values were below 0.05, 4.32% ranged between 0.05 and 0.15, and 0.07% were greater than 0.15, indicating limited genetic differentiation between the two populations (Figure 3b).

#### 2.3.2. *Leizhou Goat* TL vs. SL Based on Fst and θπ Selected Regions

In this study, the genomes of Leizhou goats in the TL group and SL group were scanned by Fst and θπ joint analysis, respectively, and the Fst values with window values of both the top 5% and θπ values of the top 5% were selected as the candidate windows for the proposed study in order to explore the candidate genes related to the leg length trait in Leizhou goats. As shown in Figure 3c, a total of 754 selection signal windows were screened in the top 5% selected region of the Fst and θπ joint screening in the SL group, among which the most selection signal windows were found on chromosome 19, with a total of 109, and the least were found on chromosomes 23 and 25, with only one. By annotating these 754 selection signal windows, a total of 804 genes were annotated. Among these genes, *B4GALT7*, *NR1D1*, *PARP2*, *SOST*, *GDF5*, *CERS3*, *EIF2AK2*, and other genes are closely related to biological processes such as chondrogenesis and osteoblast development. Among the top 5% selected regions jointly screened by Fst and θπ, a total of 407 selection signal windows were screened in the TL group, among which the selection windows located on chromosome four were the most numerous, with 39, and a total of 420 genes were annotated by annotation of these 407 selection windows. In addition, the selected regions of the two groups had two commonly selected genes, *CFAP221* and *AMBRA1*.

#### 2.3.3. Functional Annotation of Strong Selection Signal Genes in the SL Group

**Figure 4:**
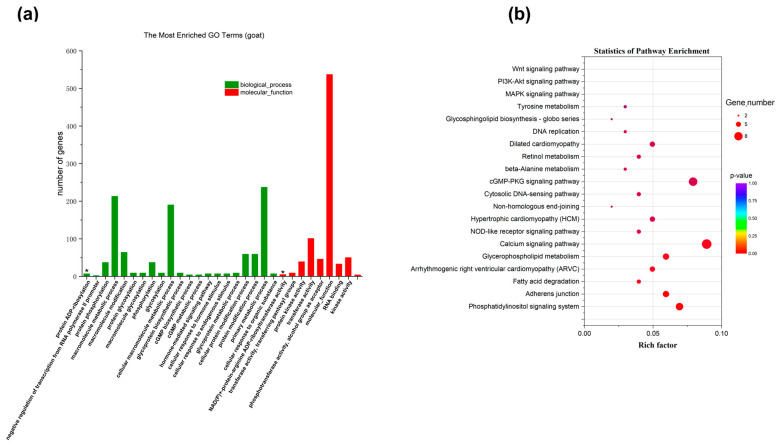
Functional enrichment analysis of selected genes in SL group was performed using GO and KEGG databases: (**a**) GO enrichment analysis of selected genes for leg length traits in Leizhou goats in SL group; (**b**) KEGG enrichment analysis of leg length trait of Leizhou goat in SL group (* indicates *p* < 0.001, suggesting a very strong significance).

To understand the function of the 804 selected genes in the SL group, GO and KEGG enrichment analyses were performed (Figure 4a). A total of 1591 GO terms were enriched, with 99 entries showing *p* < 0.05. These entries were primarily associated with biological processes such as protein ADP-ribosylation, transcription regulation, and protein phosphorylation. KEGG analysis identified 228 enriched pathways, with the most significant being circadian rhythm, calcium signaling, and glycosaminoglycan biosynthesis pathways. Additionally, key growth pathways such as the MAPK, PI3K-AKT, and WNT signaling pathways were identified (Figure 4b).

#### 2.3.4. Functional Annotation of Strong Selection Signal Genes in the TL Group

**Figure 5:**
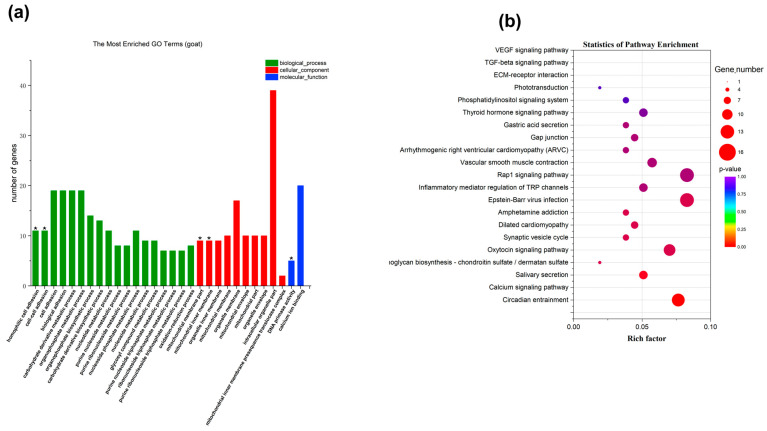
Functional enrichment analysis of selected genes in TL group was performed using GO and KEGG databases: (**a**) GO enrichment analysis of selected genes for leg length traits of Leizhou goats in TL group; (**b**) KEGG enrichment analysis of selected genes for leg length traits of Leizhou goats in TL group (* indicates *p* < 0.001, suggesting a very strong significance).

In the TL group, GO enrichment analysis identified 1338 terms, with 151 showing *p* < 0.05 (Figure 5a). These terms were associated with biological processes like cell adhesion and metabolic processes. KEGG enrichment analysis identified 205 signaling pathways, with the phosphatidylinositol signaling system, calcium signaling pathway, and ECM-receptor interaction among the most significant. These pathways are closely related to animal growth and bone development (Figure 5b).

### 2.4. GWAS Analysis of Leg Length Traits in Leizhou Goats

**Figure 6:**
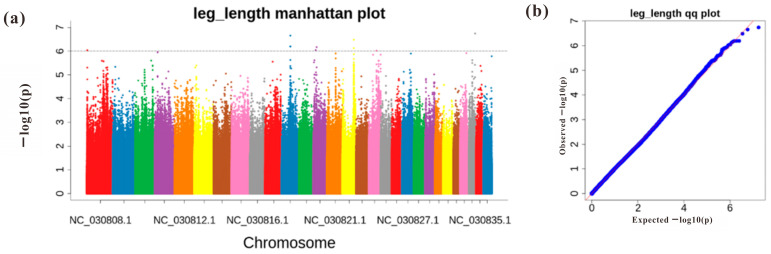
(**a**): Genome-wide association analysis of leg length in Leizhou goats: (**b**): Manhattan and Q-Q plots (Color changes in the plot are where chromosomes change).

GWAS identified 11 SNP loci significantly associated with leg length traits (Figure 6). Three loci were located in the intronic regions of the *TIAM1*, *MALRD1*, and *MCPH1 genes*, while eight were in intergenic regions. The most significant SNPs were located in the intron of the *MCPH1 gene* on chromosome 27.

### 2.5. Association Analysis of Candidate Gene Polymorphisms and Leg Length Traits

#### 2.5.1. DNA Mixed Pool Detection of Candidate SNP Polymorphisms

**Figure 7:**
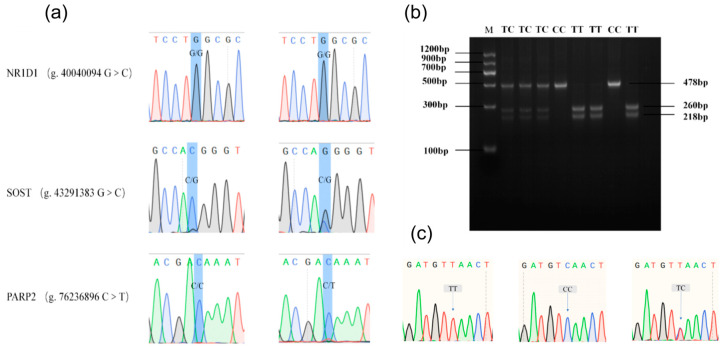
(**a**) Polymor-208 phisms of candidate SNPs for DNA pooling detection (only some of them are listed); (**b**) 209 NC_030818.1 53666634 T > C) electropherogram after digestion; (**c**) NC_030818.1 (g. Sequencing re-210 sults of 53666634 T > C) locus.

Sanger sequencing results showed that mutation sites of NC_030818.1 (g. 53666634 T > C), *SOST* (g. 43291383 G > C), *SHBG* (g. 27088465 A > G), *CAPN12* (g. 49371337 C > T), and *FRMD5* (g. 55009283 C > T) had multiple peak shapes, indicating that these candidate loci might be polymorphic in the population. In contrast, the mutation sites of *NR1D1* (g. 40,040,094 G > C), *NFKBIB* (g. 49514296 C > T), and *PARP2* (g. 76236896 C > T) had a single peak shape, suggesting that they might not be polymorphic in the population (Figure 7a). Therefore, Subsequently, only the NC_030818.1 (g. 53666634 T > C), *SOST* (g. 43291383 G > C), *SHBG* (g. 27088465 A > G), *CAPN12* (g. 49371337 C > T), and *FRMD5* (g. 55009283 C > T) mutation sites were grouped body polymorphism analysis.

#### 2.5.2. Candidate Gene SNP Site Genotyping

After conducting an analysis using Snapgene (5.0.5) software, it was found that the mutations in NC_030818.1 (g. 53666634 T > C), *SHBG* (g. 27088465 A > G), *CAPN12* (g. 49371337 C > T), and *FRMD5* (g. 55009283 C > T) were capable of affecting the enzymatically cleaved loci, and the mutations in *SOST* (g. 43291383 G > C) mutations had no effect on the enzymatic locus, so subsequent experiments were performed using the enzymatic method for NC_030818.1 (g. 53666634 T > C), *SHBG* (g. 27088465 A > G), *CAPN12* (g. 49371337 C > T), *FRMD5* (g. 55009283 C > T) genotyping. The amplification products were digested by restriction endonuclease and detected by 2% agarose gel electrophoresis, which showed three genotypes (Figure 7b). Sanger sequencing was performed on these three types of PCR products, and the results showed that the fragment sequences of the three genotypes were consistent with the sequences on NCBI, and the mutation types of the bases were the same as those in the enzyme digestion results (Figure 7c), indicating that the PCR products were amplified accurately and the enzyme digestion sites were selected correctly, which ensured the reliability of the subsequent analysis.

#### 2.5.3. Candidate Gene Polymorphism Analysis

The genetic parameters of the SNP loci of the candidate genes are shown in Table 4, from which it can be seen that the PICs of NC_030818.1 (g. 53666634 T > C) and SHBG (g. 27088465 A > G) were between 0.25 and 0.5, which exhibited moderate frequency, while the PICs of CAPN12 (g. 49371337 C > T) and FRMD5 (g. 55009283 C > T) all had polymorphic information content (PIC) less than 0.25, showing lower frequency. The degree of purity and heterozygosity of NC_030818.1 (g. 53666634 T > C) and SHBG (g. 27088465 A > G) were both around 0.5, indicating that these two alleles were more uniformly distributed in the population of Leizhou goat.

#### 2.5.4. Association Analysis of NC_030818.1 (g. 53666634 T > C) Gene Polymorphism and Leg Length

SPSS 26.0 software was used to analyze the association between the NC_030818.1 (g. 53666634 T > C) variant locus and the leg length of different genotypes of Leizhou goats, and it can be seen in Table 5 that the mean value of the leg length of the Leizhou goats with the CC genotype was 24.156 cm, the mean value of the leg length of the Leizhou goats with the TC genotype was 28.278 cm, and the mean value of the leg length of the Leizhou goats with the TT genotype was 30.408 cm. The mean value of the leg length of Leizhou goats was 30.408 cm. In terms of leg length of Leizhou goats, the leg length of TT genotype was longer than that of CC genotype by 6.352 cm, with a significant difference (*p* < 0.05).The leg length of TT genotype was longer than that of TC genotype by 2.13 cm, with a significant difference (*p* < 0.05), and the leg length of TC genotype was longer than that of CC genotype by 4.222 cm, with a significant difference (*p* < 0.05).

## 3. Discussion

### 3.1. Characterization of the Distribution of Genetic Variation in the Genomes of TL and SL Groups of Leizhou Goats

In this study, we identified a total of 8,641,229 high-quality SNP sites through whole-genome resequencing of tall and short-legged Leizhou goats. Our analysis revealed that 5,455,335 (63.13%) of these SNPs were intergenic, while 2,976,338 (34.44%) were intronic. These proportions are consistent with previous findings in Hainan black goats by Chen et al. [8], where the majority of SNP variants were also found in non-coding regions. Our results suggest that the large genomic segments occupied by intergenic and intronic regions contribute to the high number of SNPs in these areas. Although intergenic regions do not code for proteins, they can give rise to non-coding RNAs that play significant roles in regulatory processes, as supported by our data and the broader literature.

Our study further indicates that non-coding RNAs, once considered ‘gene noise’ are essential for various biological functions, and their dysregulation can be linked to disease susceptibility. For example, we observed that certain SNPs in non-coding regions of Leizhou goats are associated with growth performance, which aligns with the reported impact of miR-1666 mutations on chicken growth [9]. This finding underscores the importance of understanding the functional implications of SNPs in non-coding regions for goat breeding programs.

Additionally, our research identified SNPs within exonic regions and the 1 kb flanking sequences of genes. These SNPs are of particular interest because they have the potential to affect protein coding and gene regulation. Our data show that mutations in exons can lead to amino acid changes, protein misfolding, or even embryonic lethality, highlighting the critical role of exon integrity in maintaining phenotypic stability [10]. Moreover, SNPs in the 1 kb upstream region, which is rich in transcription factor binding sites, can modulate gene expression levels, while those in the downstream region can influence transcriptional termination and mRNA processing. These findings are crucial for understanding the molecular basis of phenotypic variation in Leizhou goats and can inform selective breeding strategies.

### 3.2. Population Genetic Structure Analysis of Leizhou Goats with TL and SL Groups

In this study, we utilized population evolutionary trees, principal component analysis (PCA), and genetic structure analysis to examine the evolutionary relationships between TL and SL Leizhou goats. Our findings suggest a genetic differentiation between these groups, though evidence of genetic mixing was also present. Notably, three individuals from the TL group, identified as TL phenotypically, appeared genetically closer to the SL group in the evolutionary tree. This finding may be attributed to genetic exchange between TL and SL goats during breeding, which is plausible given that both groups share a common farm origin. This observed genetic mixing could be advantageous, as gene flow might help maintain genetic diversity, thereby potentially contributing to the adaptability and resilience of Leizhou goats. Moreover, the minor genetic differentiation observed likely reflects their shared ancestry, which suggests that while selective breeding for distinct leg length may drive differentiation, shared origins and interbreeding constrain it.

### 3.3. Selected Genes and Functional Analysis of TL and SL Groups in Leizhou Goats

In this study, the TL and SL groups of Leizhou goats were jointly analyzed using Fst and θπ for population selection elimination, and a total of 1161 selected regions were screened in the top 5%, and a total of 1222 selected genes were obtained from the gene annotation of these selected windows, of which 420 genes were screened in the TL group, and 804 genes were screened in the SL group. The common genes screened in both groups were *CFAP221* and *AMBRA1*. The *CFAP221* gene encodes a protein involved in the regulation of cilia and flagellum assembly, stability, and motility. It has been shown that the *CFAP221* gene regulates the assembly and motility of cilia and flagella. Mutations in this gene have been associated with primary ciliary dyskinesia, abnormal spermatogenesis, and embryonic death in mice models [11]. Studies on *CFAP221* in goats have not yet been reported. The protein encoded by the *AMBRA1 (AutophagyAndBeclin1Regulator1)* gene is a key regulator in autophagy, which is highly conserved in vertebrates and has a wide range of biological functions, including metabolism, cell death, and cell division [12]. Its mutation has been implicated in nervous system disorders and carcinogenesis, as well as skeletal muscle maintenance and autism in human and mouse studies [13]. Therefore, mutations in the *AMBRA1* gene may affect autophagic activity in the animal organism and thus participate in the regulation of animal life activities, but whether it can be used as a molecular marker gene for tall and short feet in Leizhou goats still needs further research and exploration.

In the top 5% of selection signals for the SL group, several genes closely associated with biological processes, such as cartilage and osteoblast development, were annotated, including *B4GALT7*, *NR1D1*, *PARP2*, *SOST*, *GDF5*, *CERS3*, and *EIF2AK2*. Animal growth and development are closely linked to skeletal growth. Mammalian bone development primarily occurs through intramembranous and endochondral ossification, with endochondral ossification being the predominant form in limb formation [14]. The *B4GALT7 gene* encodes a protein that belongs to the β-1,4-galactosyltransferase family, which is specific for the donor substrate UDP-galactose [15]. Numerous studies indicate that mutations in the *B4GALT7 gene* can lead to abnormal skeletal development, resulting in conditions such as short stature and dwarfism [16]. Furthermore, mutations in *B4GALT7* have been linked to dwarfism in Frisian horses, with the specific mutation *B4GALT7*(g. 4535550 C > T) leading to a significant decrease in *B4GALT7* expression and resulting in shortened limbs [17]. In zebrafish, the knockdown of *B4GALT7* results in the loss or severe deformation of craniofacial cartilage and bone structures [18]. Thus, *B4GALT7* plays a key role in early cartilage, bone, and muscle development, and its mutations may influence skeletal development in Leizhou goats, affecting leg length traits.

*NR1D1*, a member of the nuclear receptor family, regulates target genes associated with various physiological processes, including autophagy, immunity, inflammation, metabolism, and multi-organ aging [19]. The *SOST* gene encodes sclerostin, primarily expressed in osteocytes, and several GWAS findings indicate that SNP sites at the *SOST* locus are significantly associated with bone mineral density and fracture susceptibility [20]. In humans, mutations in the *SOST* gene are strongly linked to osteoporosis, particularly in postmenopausal women [21]. *GDF5*, a member of the BMP family, is highly expressed in the surface layer of articular cartilage. The *GDF5* gene is a key risk locus for osteoarthritis, with *GDF5*-deficient mice exhibiting abnormal joint development, underscoring its critical role in joint development and homeostasis [22]. Numerous studies have found that *GDF5* mutations correlate with susceptibility to human osteoarthritis [23], and the TT genotype of *GDF5* SNP rs143383 increases susceptibility to knee arthritis in northern Mexico [24]. Furthermore, molecular marker association analysis in cattle has linked SNPs in the *GDF5* gene to domestic cattle size traits, indicating that the *GDF5* SNP may serve as a valuable genetic marker for breeding [25]. These findings suggest that mutations in these genes may influence leg length traits in Leizhou goats by affecting skeletal development.

Additionally, several genes associated with bone development were identified in the TL group, including *SP1* and *KIF7*. The protein encoded by *SP1* is a zinc finger transcription factor that regulates various physiological processes, including cell growth, apoptosis, differentiation, and immune response, by binding to GC-rich promoter regions. *SP1* has been shown to regulate the expression of the *bone morphogenetic protein 2 (BMP2)* gene [26], which is crucial for osteoblast differentiation. Conditional knockout of *BMP2* in mice results in reduced bone mass and skeletal defects [27]. Therefore, SNPs in SP1 may influence *BMP2* protein expression, thereby regulating osteoblast differentiation during the growth and development of Leizhou goats. Moreover, *SP1* can bind to the PPARα promoter region, affecting its activity and thus regulating bone metabolism [28]. Polymorphisms in SP1 binding sites within the *COL1A1* gene are significantly associated with clinical phenotypes, including collagen disease and osteogenesis imperfecta (OI), with mutations at the rs1800012 site linked to bone mineral density in women aged 45 years and older [29]. Proper regulation of chondrocyte proliferation and differentiation is essential for normal skeletal growth. During endochondral bone development, signaling pathways regulate growth plate chondrocyte differentiation, and disruptions can lead to skeletal dysplasia and short stature [30]. The Hedgehog (Hh) signaling pathway plays a vital role in bone growth regulation. KIF7, a kinesin motor protein, is involved in Hh signaling, and KIF7-null embryos exhibit mild ectopic activation of the Hh pathway. KIF7 limits the inhibitory effect of SUFU, promoting Hh signal transduction in growth plate chondrocytes [31]. Although this study has identified many candidate genes associated with leg length traits in Leizhou goats, it remains to be determined whether these genes are critical within the population. Nevertheless, the candidate genes identified here provide valuable references for breeding tall and short Leizhou goats in practical applications.

Functional enrichment analysis of the selected genes revealed associations with various signaling pathways related to bone development, including glycosaminoglycan biosynthesis (cartilage sulfate/skin sulfate), the WNT signaling pathway, and the Hedgehog signaling pathway. Chondroitin sulfate, a major component of bone and connective tissues, promotes chondrocyte proliferation and differentiation, regulating skeletal growth and repair processes. Furthermore, chondroitin sulfate influences the synthesis and degradation of the extracellular matrix, maintaining bone tissue health and facilitating normal bone formation and function. During endochondral ossification, Ihh production by chondrohypertrophic chondrocytes signals perichondrial cells to induce osteoblast differentiation. In endochondral bone, the Hedgehog signaling pathway initiates osteoblast differentiation by inducing WNT ligand expression. These signaling molecules ultimately regulate osteocyte proliferation and differentiation through transcription factor expression [32]. WNT proteins are key regulators of differentiation and activity in both mouse and human osteoblasts. Activation of WNT signaling by a single allele of *Sfrp1*, *SOST*, or *Dkk1* increases osteoblast numbers and activity, while inhibition of WNT ligand secretion suppresses bone formation in mice [33]. β-Catenin, a core protein in the WNT signaling pathway, collaborates with TCF1 to directly stimulate Runx2 transcription, regulating skeletogenesis [34]. The WNT signaling pathway also activates PKCD and mTORC1, promoting protein synthesis, bone formation, and osteoblast differentiation [35]. Thus, genes subject to selection in the dwarf population may regulate glycosaminoglycan biosynthesis, the cartilage sulfate/skin sulfate pathway, WNT signaling, and the Hedgehog signaling pathway.

Additionally, GO and KEGG functional enrichment analyses of selected genes indicated involvement in the Notch signaling pathway, vascular endothelial growth factor (VEGF) signaling pathway, and TGF-β signaling pathway, all of which are critical for animal growth and skeletal development. The Notch signaling pathway regulates the differentiation and function of osteoblasts and osteoclasts, playing a key role in skeletal development, chondrogenesis, osteogenesis, and osteoclastogenesis [36]. Notch1 inhibits osteoblast formation by suppressing Runt-associated transcription factor 2 and cytoplasmic β-catenin, while Notch2 induces osteoclast differentiation, increasing bone resorption and decreasing bone mass [37]. The Notch signaling pathway is also associated with the growth, differentiation, and apoptosis of bone marrow stem cells (BMSCs), promoting the formation of immature osteoblasts while inhibiting the maturation of terminal osteoblasts. Angiogenesis is essential for bone production during skeletal development, with HIF-1α and the HIF-responsive gene VEGF being key factors linking osteogenesis and angiogenesis [38]. VEGFA promotes the maturation of bone marrow osteoclasts in vitro and induces the differentiation of bone marrow osteoclasts into mature osteoclasts, thus playing a role in skeletal development. The TGF-β signaling pathway is crucial for multiple cellular life activities and essential for bone formation during mammalian growth. It interacts with other signaling pathways, including MAPK, WNT, Hedgehog, Notch, and FGF pathways, to regulate skeletal development. Notably, knockdown of the *TGFBR2* gene within the TGF-β signaling pathway results in abnormal bone development, including shortened limbs and defects in long bone and mandibular formation. Therefore, mutations in the selected genes may affect Notch signaling, VEGF signaling, TGF-β signaling, and other pathways, ultimately influencing the development of goat bones and affecting leg length traits.

### 3.4. GWAS Association Analysis of Leg Length Traits in Leizhou Goats

In this study, the association analysis of leg length data from 30 Leizhou goats identified 11 SNP loci significantly associated with leg length traits, of which three were annotated to the *TIAM1*, *MALRD1*, and *MCPH1* genes. The *TIAM1* gene encodes proteins that play important roles in cell signaling and migration. *TIAM1* is a guanylate nucleotide exchange factor that primarily mediates the activation of the Rac small G protein. Rac, a member of the Rho family of GTPases, plays a crucial role in cytoskeletal remodeling, cell migration, cell adhesion, and other processes [39]. By promoting Rac activation, *TIAM1* regulates cytoskeletal dynamics, thereby influencing cell polarity, adhesion, and migratory capacity [40]. This enables *TIAM1* to play an important role in tumor metastasis and invasion processes. Additionally, *TIAM1* is involved in neuronal formation and the regulation of synaptic plasticity, both of which are crucial for the development and function of the nervous system [41]. In pigs, several studies have identified the *TIAM1* SNP locus as a potential marker for economically important traits, with significant correlations found between the *TIAM1* gene SNP and residual feeding as well as litter size in Chinese native pigs [42]. However, no studies have reported on this gene in goats.

The *MALRD1* gene encodes a protein containing multiple MAM (meprin-A5 protein tyrosine phosphatase mu) and LDLRA2 (low-density lipoprotein receptor A2) domains, which regulate FGF19 production in human intestinal cell lines, thereby modulating bile acid levels, inhibiting gluconeogenesis and lipogenesis, and promoting glycogen synthesis [43]. Within-population GWAS and cross-population meta-analyses of two different strains of Large White pigs indicated that mutations in the *MALRD1* gene were significantly associated with gestation length [44]. Moreover, a genome-wide association study in white Australians with type 2 diabetes revealed that mutations in *MALRD1* (rs12267418) were significantly associated with diabetic macular edema.

The *MCPH1* gene encodes a DNA damage response protein involved in maintaining the inhibitory phosphorylation of cyclin-dependent kinase 1 (CDK1) and plays a crucial role in G2/M checkpoint arrest [45]. The *MCPH1* gene encodes a DNA damage response protein involved in maintaining the inhibitory phosphorylation of cyclin-dependent kinase 1 (CDK1) and plays a crucial role in G2/M checkpoint arrest [46]. Numerous studies have demonstrated that mutations in the *MCPH1* gene are associated with type 1 primary microcephaly and precocious chromosome condensation syndrome [47]. Additionally, the *MCPH1* gene acts as a repressor of hTERT, mediating the DNA damage response and maintaining chromosomal integrity, and its downregulated expression is linked to the occurrence of cervical cancer [48].

## 4. Materials and Methods

### 4.1. Sample Collection and DNA Extraction

One hundred and two ewes of similar ages and body conditions and with consistent feeding and management conditions, good physical condition, and no disease record were selected from Leizhou Goat Farm in Zhanjiang City, Guangdong Province. After body measurements, 10 mL of jugular vein blood was collected from Leizhou goat ewes using disposable vacuum blood collection tubes containing EDTA-K2 as an anticoagulant. Following collection, the tubes were gently inverted to ensure thorough mixing of the blood with the anticoagulant. The samples were then immediately transferred to liquid nitrogen for preservation and transportation. Upon arrival at the laboratory, they were stored at −80 °C. Blood samples from 15 Leizhou goats (Table A1) with the longest leg lengths and 15 with the shortest leg lengths were sent to Beijing Novogene Co., Ltd. (Beijing, China). for whole-genome resequencing. Genomic DNA extraction and quality testing were also performed by Beijing Novogene Co., Ltd. The DNA quality assessment methods included (1) agarose gel electrophoresis to check for contamination by proteins, RNA, or signs of degradation; (2) nanodrop spectrophotometry to measure the OD260/280 ratio and assess protein or RNA contamination; and (3) Qubit fluorometry for precise DNA concentration quantification. Only DNA samples with OD260/280 ratios between 1.8 and 2.0, and DNA concentrations of 1.5 μg or higher, were selected for library construction and sequencing.

### 4.2. DNA Library Construction and Quality Testing

Qualified DNA samples were randomly fragmented into 350 bp lengths using a Covaris ultrasonic crusher. The fragmented DNA underwent end repair, polyA tailing, adapter ligation, purification, and PCR amplification to complete library preparation. The NEBNext^®^ Ultra™ II DNA Library Prep Kit (New England Biolabs, Ipswich, MA, USA) was used for library construction. Following this, the product was quantified using Qubit 3.0 and diluted to a concentration of 1 ng/μL. The Agilent 5300 and Q-PCR were then employed to accurately measure the effective concentration of the library, ensuring it exceeded 2 nM. Finally, the library was subjected to PE150 sequencing using the Illumina platform, with a sequencing depth of 10×.

### 4.3. Sequencing Data Quality Control and Mapping

Raw image data files obtained from the Illumina platform were converted into raw sequenced reads, known as “raw data”, using CASAVA (1.8.2) BaseCalling analysis. Since the raw data cannot be directly analyzed, it underwent a series of filtering processes before being used for further analysis. The filtering criteria included (1) removing read pairs with adapter sequences, (2) discarding reads where the proportion of ‘N’ content exceeded 10%, and (3) excluding reads where more than 50% of the bases had a quality score of Q ≤ 5. After applying these filtering conditions, “clean data” were obtained, and all subsequent analyses were conducted on these clean reads.

Paired-end reads were mapped to the goat reference genome using the BWA software (https://ftp.ncbi.nlm.nih.gov/genomes/all/GCF/001/704/415/GCF_001704415.1_ARS1/, accessed on 19 July 2022), with the command “bwa mem -*t* 4 -k 32 -M”. After mapping, potential PCR duplicates were removed using the “rmdup” command in SAMTOOLS, retaining only the read pairs with the highest mapping quality.

### 4.4. SNP Detection and Annotation

Sequencing data were aligned for SNP calling at the population scale using the Bayesian approach in SAMTOOLS, then genotype likelihoods were calculated based on the number of reads for each individual at each genomic position, and allele frequencies in the samples were calculated using the Bayesian approach. Individual SNPs were identified using SAMTOOLS (Dp4-miss0.1-maf0.05). Then, in order to retain high-quality SNPs to exclude SNP identification errors due to mismapping or INDELS, a filtering process was required for the raw SNPs, with the filtering conditions that (1) the quality value of SNPs was ≥20, and (2) the number of read supports of SNPs was ≥4. The filtered high-quality SNPs were annotated using ANNOVAR based on the goat DNA reference genome. According to the genome annotation, SNPs were categorized into exonic regions (overlapping with coding exons), intronic regions (overlapping with introns), splice sites (within 2 bp of splice junctions), upstream and downstream regions (within a 1-kb region upstream and downstream of the transcriptional start site), and intergenic regions. SNPs on coding exons were further categorized into synonymous SNPs (which do not cause amino acid changes) and non-synonymous SNPs (which cause amino acid changes), as well as SNPs that make based on the gain or loss of a stop codon.

### 4.5. Population Genetic Structure Analysis

In this study, the neighbor-joining method was employed to construct the phylogenetic tree. After SNP detection, the individual SNPs obtained were used to calculate the genetic distance between populations. The *p*-distance between two individuals *i* and *j* was calculated using Equation (1):

(1) $$D_{ij}=\frac{1}{L}\sum_{i-1}^{L}d_{ij}^{\left(1\right)}$$

where *L* is the length of the region with high-quality SNPs. If the allele at position 1 is A/C, the genetic distance function is defined as:

(2) $$d_{ij}^{\left(1\right)}=\left\{\begin{array}{c} 0,\;\;\;\;\;\;\;\;\;\;\;\;If\;the\;genotypes\;of\;two\;individuals\;are\;AA\;and\;AA\\ 0.5,\;\;\;\;\;\;\;\;\;\;\;If\;the\;\;genotypes\;of\;two\;individuals\;are\;AA\;and\;AC\\ 0.5,\;\;\;\;\;\;\;\;\;\;\;If\;the\;\;genotypes\;of\;two\;individuals\;are\;AC\;and\;AC\\ 1,\;\;\;\;\;\;\;\;\;\;\;\;If\;the\;genotypes\;of\;two\;individuals\;are\;AA\;and\;CC \end{array}\right.$$

The distance matrix was computed using Treebest-1.9.2 software, and a phylogenetic tree was constructed using the neighbor-joining method. Bootstrap values were obtained after performing 1000 iterations.

In this study, principal component analysis (PCA) was performed as follows: the SNPs at position *k* of individual *i,* are denoted by [0–2], which is zero if individual *i* is purely homozygous for the reference allele, one if heterozygous, and two if individual *i* is purely homozygous for the non-reference allele. m is a matrix containing the n × S matrix of standard genotypes:

(3) $$d_{ik}^{\prime }\;=\;\left(d_{ik}-E\left(d_{k}\right)\right)/\sqrt{E\left(d_{k}\right)\times \left(1-E\left(d_{k}\right)/2\right)/2}$$

In Equation (3), *E* (*d_k_*) is the mean of *d_k_*, and the covariance n × n matrix of individual samples is calculated by X = MMT/S. The PCA analysis was performed using GCTA (1.24.2) software, and finally, the values of the first three PCs were taken for plotting. In this study, PLINK [49] was used for population structure analysis, first creating a PLINK input file and then using ADMIXTURE (1.23) software [50] to construct population genetic structure and population lineage information.

(PLINK (https://s3.amazonaws.com/plink1-assets/1.07/plink1_linux_x86_64.zip), accessed on 13 November 2024).

### 4.6. Population Selection Elimination Analysis

In this study, the whole-genome of the Leizhou goat TL group and SL group was scanned with the sliding window method, and three methods, Fst, θπ, and Fst and θπ, were used to detect the selection signal of the Leizhou goats. The window size was first selected based on the density of SNPs on the genome, and it was found that the number of SNPs on the window tended to stabilize from 100 kb through analysis, so the window length for selective elimination analysis was selected to be 100 kb.

Population genetic differentiation index Fst is an index used to measure the degree of genetic differences between different populations, which plays an important role in understanding the genetic structure, kinship, and genetic flow between different populations. The closer it is to one, the larger the genetic differences between populations, the higher the degree of differentiation, and the more distant the kinship. In this study, vcftools_v0.1.14 software was used to calculate the Fst value for each window, assessing the genetic structure of the tall- and short-legged Leizhou goat populations.

Nucleotide polymorphism θπ is often used in animal genetic breeding to assess genetic diversity, QTL localization, kinship analysis, and population structure analysis, and is a positive guide for selecting parents, improving quantitative traits, and avoiding inbreeding and selection bias. The study used vcftoolsv_0.1.14 software [51] to calculate the θπ values of different subgroups of Leizhou goats with 100 kb for each window and 50% overlap as the step size. θπ values that deviate more from one represent a higher degree of being subjected to selection.

As an effective method for detecting selection elimination regions, the joint screening of Fst and θπ can jointly screen for stronger selection signals, which in turn facilitates the screening of candidate genes for target traits. In this study, the top 5% of windows for both Fst and θπ were intersected to identify common selection regions. These regions were then annotated to identify candidate genes potentially associated with leg length traits in Leizhou goats.

### 4.7. Gene Functional Enrichment Analysis

In this study, we first mapped the genes annotated to the Fst and θπ selected regions into the GO database by mapping them to the Fst and θπ selected regions, then we calculated how many genes were mapped to each entry, and finally, we calculated the *p*-value values of the mapped entries using hypergeometric test analysis. The KEGG pathway enrichment analysis was performed on the genes annotated to the Fst and θπ selected regions using the KOBAS website to analyze the potential biological functions of the annotated genes in the selected regions [52].

### 4.8. Genome-Wide Association Analysis

In this study, data from 30 Leizhou goats were obtained based on whole-genome resequencing and analyzed using GWAS using GEMMA (7 March 2010) [53], a genome-wide efficient mixed-model association software package. For MLM analysis, Equation (4) was used:

(4) $$y=X\alpha +Z\beta +W_{\mu }+e$$

In Equation (4), *y* represents the phenotype, *X* represents the genotype, S is the structure matrix, and *Z* is the indicator matrix of SNP. *α* is the estimated parameter of fixed effect, *β* is the effect of SNP, *W* is the indicator matrix of random effect, *μ* is the predicted random individual, and *e* is the random residuals. Statistical analysis was performed using the GEMMA package. A significant (0.05/N) *p*-value threshold was set to control for genome-wide type 1 error rates (N is the number of SNP involved in the analysis: 8,631,706).

### 4.9. Candidate SNP Gene Polymorphisms and Their Leg Length Trait Association Analysis

Sanger sequencing and enzymatic digestion methods were employed to examine the candidate SNP loci in an expanded goat population. The experimental procedure encompassed DNA extraction, primer design, PCR amplification, enzymatic digestion of PCR products, and subsequent Sanger sequencing.

(1) Blood genomic DNA extraction

DNA was extracted from the blood of 250 Leizhou goats with recorded body size measurements. Blood genomic DNA was extracted from the collected samples using the universal DNA extraction kit of Meiji Bio, and the operation steps were as follows: (1) 20 μL of proteinase K was added to the centrifuge tube in advance, then 200 μL of anticoagulated blood was added, vortexed, and shaken; (2) 200 μL of buffer was added after the above steps and vortexed and shaken, and then incubated at 70 °C for 10 min; (3) after the above steps, 200 μL of anhydrous ethanol was added, and vortexed and shaken; (4) the above reaction solution was transferred to the HiPureDNAMiniColumn1 filtration column, and centrifuged at 10,000× *g* for 1 min; (5) 500 μL of ethanol-diluted BufferGW1 was added to the filtration column and centrifuged at 10,000× *g* for 1 min; (6) 650 μL of ethanol-diluted BufferGW2 was added and centrifuged at 10,000× *g* for 1 min; (7) the filtrate was discarded and centrifugation was continued at 10,000× *g* for 3 min; (8) 50 μL of BufferAE as added at 70 °C to the filtration column, left at room temperature for 3 min, and then centrifuged at 10,000× *g* for 1 min; and (9) an ultra-micro spectrophotometer was used to perform concentration and OD260/280 detection, and DNA samples were randomly drawn for agarose gel electrophoresis to analyze the DNA integrity (Figure 7a).

(2) Primer design

Primers for the SNP regions were designed using the NCBI Pick primer tool. The primer sequences are listed in Table 6.

(3) PCR amplification

Primers were designed based on 600 bp regions upstream and downstream of the SNP mutation sites using NCBI resources. PCR amplification of Leizhou goat genomic DNA was performed, and the PCR products were analyzed via agarose gel electrophoresis (Figure 7b). PCR amplification of the extracted DNA was performed using the two × SuperTaq PCR StarMix from Kangrun Bio (Guangzhou, China). The reaction conditions and program are shown in Table 7 and Table 8.

(4) PCR product digestion and Sanger sequencing

The PCR products were digested with the appropriate restriction enzyme based on the SNP mutation site, and genotypes were determined from the digestion products. For the fragments with no suitable endonuclease at the SNP mutation site, the products were genotyped by Sanger sequencing, which was carried out by Sangon Biotech (Shanghai, China).

(5) Select SNP loci

SNP loci with strong correlations between the selective sweep analysis and GWAS were selected, and then the Leizhou goat population was expanded for association analysis. The selected SNP loci included NC_030818.1 (g. 53666634 T > C), *NR1D1* (g. 40040094 G > C), *PARP2* (g. 76236896 C > T), *SOST* (g. 43291383 G > C), *NFKBIB* (g. 49514296 C > T), *SHBG* (g. 27088465 A > G), *CAPN12* (g. 49371337 C > T), and *FRMD5* (g. 55009283 C > T) (Table A2).

(6) DNA mixed pool detection of candidate SNP polymorphisms

DNA samples of 30 Leizhou goats were randomly selected to make DNA mixing pools, and a total of three DNA mixing pools were made. Using the DNA mixed pool as a template, PCR amplification was carried out using specific primers, and the amplified products were sequenced by Sanger sequencing.

(7) Candidate gene SNP site genotyping

Using goat DNA as a template, specific primers were used to amplify the fragments where the mutation sites of the candidate genes were located, after which genotyping was carried out by enzymatic digestion.

### 4.10. Data Processing and Analysis

The leg length data of Leizhou goats were calculated as the difference between body height and chest depth. Statistical analysis was performed using the General Linear Model (GLM) in SPSS 26.0. The analysis utilized a constructed unit point effect model to assess the relationship between molecular marker loci and leg length in Leizhou goats. Multiple comparisons between means were conducted using LSD and Dunnett’s T3 methods to evaluate the effect of genotype on leg length. Results are expressed as “mean ± standard deviation” (Mean ± SD). The linear model used for statistical analysis is represented by Equation (5):

(5) $$y_{jkl}=\mu +G_{k}+e_{j\kappa l}$$

where *y_jkl_* is the leg length value of the Leizhou goat, *μ* is the population mean, *G_k_* is the fixed effect of the kth genotype, and *e_jkl_* is the random residual effect.

## 5. Conclusions

(1) A total of 8,641,229 high-quality SNPs were identified in 30 Leizhou goats using whole-genome resequencing. Eight candidate genes that might be related to leg length traits in Leizhou goats were screened using selective elimination analysis, including *B4GALT7*, *NR1D1*, *PARP2*, *SOST*, *GDF5*, *EIF2AK2*, *SP1*, and *KIF7*.
(2) NC_030818.1 (g. 53666634 T > C) and *SHBG* (g. 27088465 A > G) showed intermediate allele frequency in the Leizhou goat population. In addition, the NC_030818.1 (g. 53666634 T > C) variant was associated with leg length traits, with shorter leg lengths in the CC type and longer leg lengths in the TT type.

## Figures and Tables

**Table 1:** Summary of sequencing data quality.

Sample	Raw Base (bp)	Clean Base (bp)	Effective Rate (%)	Error Rate (%)	Q20 (%)	Q30 (%)	GC Content (%)
TL1	27,843,832,200	27,649,102,800	99.30	0.03	97.64	93.34	43.72
TL2	30,256,776,900	29,683,815,300	98.11	0.03	96.91	91.86	47.05
TL3	29,662,381,500	29,443,012,200	99.26	0.03	97.37	92.39	43.55
TL4	27,814,504,200	27,618,588,900	99.30	0.03	97.34	92.65	43.62
TL5	28,073,487,000	27,882,138,300	99.32	0.03	97.55	93.10	43.30
TL6	26,578,760,100	26,406,888,300	99.35	0.03	97.44	92.85	43.27
TL7	30,786,686,700	30,546,573,900	99.22	0.03	97.16	92.28	44.21
TL8	34,228,209,600	33,967,403,700	99.24	0.03	97.42	92.84	43.54
TL9	29,552,629,200	29,336,811,600	99.27	0.03	97.53	92.97	43.06
TL10	28,615,713,000	28,427,135,400	99.34	0.03	96.86	91.53	43.52
TL11	30,323,067,600	29,927,976,600	98.70	0.03	97.23	92.77	46.14
TL12	32,162,967,000	31,930,486,500	99.28	0.03	96.74	91.24	43.05
TL13	29,870,390,100	29,705,223,900	99.45	0.03	97.05	91.69	42.67
TL14	30,049,663,200	29,826,423,900	99.26	0.03	97.29	92.34	43.27
TL15	27,483,879,000	27,272,088,600	99.23	0.03	97.34	92.62	42.73
SL1	28,270,083,600	27,854,524,800	98.53	0.03	96.83	91.94	46.79
SL2	30,463,497,900	30,244,026,000	99.28	0.03	97.19	92.12	43.36
SL3	32,409,471,900	32,156,407,800	99.22	0.03	97.48	92.96	43.20
SL4	28,160,716,800	27,989,217,600	99.39	0.03	97.20	92.17	43.16
SL5	27,703,572,000	27,501,890,100	99.27	0.03	97.38	92.67	42.15
SL6	28,712,619,600	28,515,085,500	99.31	0.03	97.44	92.83	41.97
SL7	27,628,634,100	27,444,917,100	99.34	0.03	97.50	92.97	42.48
SL8	27,288,080,700	27,118,727,100	99.38	0.03	97.25	92.40	42.54
SL9	29,785,053,300	29,587,171,500	99.34	0.03	97.26	92.38	42.02
SL10	28,735,499,100	28,576,833,000	99.45	0.03	97.39	92.67	41.86
SL11	26,732,475,300	26,567,756,400	99.38	0.03	97.62	93.23	41.90
SL12	30,159,567,600	29,980,689,900	99.41	0.03	97.34	92.57	41.73
SL13	26,976,734,700	26,800,857,300	99.35	0.03	97.36	92.31	41.89
SL14	26,850,299,400	26,678,291,100	99.36	0.03	97.40	92.72	42.22
SL15	27,713,181,000	27,538,027,500	99.37	0.03	97.49	92.91	42.18

Note: Q20: effective rate: ratio of clean data to raw data after filtering; percentage of bases with a mass value of more than 20 (error rate of less than 1%); Q30: percentage of bases with a mass value of more than 30 (error rate below 0.1%); GC content: the proportion of bases G and C.

**Table 2:** Sequencing depth and coverage statistics.

**Sample**	**Mapped Reads**	**Total** **Reads**	**Mapping Rate (%)**	**Average Depth (X)**	**Coverage_1X**	**Coverage_4X**
TL1	189,132,200	188,528,943	0.9968	8.12	0.9439	0.8355
TL2	189,271,354	188,667,959	0.9968	8.02	0.9452	0.8448
TL3	192,572,802	191,915,407	0.9966	8.01	0.9447	0.8461
TL4	191,883,874	191,247,671	0.9967	7.95	0.9454	0.8431
TL5	178,793,644	178,142,151	0.9964	7.05	0.9440	0.8203
TL6	183,423,434	182,712,676	0.9961	7.51	0.9416	0.8054
TL7	184,470,504	183,775,701	0.9962	7.7	0.9417	0.8101
TL8	189,772,704	189,135,452	0.9966	8.04	0.9455	0.8672
TL9	200,045,044	199,368,412	0.9966	8.4	0.9442	0.8437
TL10	177,627,374	176,935,396	0.9961	7.87	0.9438	0.8305
TL11	188,357,116	187,702,096	0.9965	7.53	0.9418	0.8203
TL12	188,983,800	188,337,891	0.9966	7.84	0.9433	0.8280
TL13	195,918,658	195,318,873	0.9969	8.34	0.9462	0.8524
TL14	195,665,504	194,946,296	0.9963	8.13	0.9456	0.8527
TL15	178,251,788	177,626,089	0.9965	7.24	0.9426	0.8213
SL1	190,994,270	190,372,600	0.9967	8.04	0.9449	0.8573
SL2	190,854,390	190,252,733	0.9968	8.02	0.9445	0.8404
SL3	184,093,670	183,481,304	0.9967	7.71	0.9448	0.8493
SL4	191,850,262	191,121,591	0.9962	7.98	0.9445	0.8491
SL5	186,145,744	185,600,708	0.9971	7.67	0.9430	0.8339
SL6	190,319,146	189,735,385	0.9969	7.91	0.9436	0.8347
SL7	182,117,432	181,769,071	0.9981	7.36	0.9400	0.7930
SL8	190,220,722	189,555,444	0.9965	8.15	0.9445	0.8611
SL9	183,563,430	182,896,188	0.9964	7.58	0.9444	0.8455
SL10	189,510,436	188,942,424	0.9970	8.10	0.9393	0.8180
SL11	192,508,250	191,930,578	0.9970	8.23	0.9457	0.8538
SL12	191,705,936	191,113,160	0.9969	8.20	0.9445	0.8539
SL13	196,962,274	196,290,642	0.9966	8.44	0.9467	0.8723
SL14	195,311,816	194,163,640	0.9941	8.50	0.9444	0.8348
SL15	194,740,420	193,861,415	0.9955	8.91	0.9466	0.8696

Note: Mapped reads: number of reads mapped to the reference (including single-ended and double-ended matches); total reads: The total number of reads for valid sequencing data; mapping rate: alignment rate, the number of reads aligned to the reference genome divided by the number of reads for valid sequencing data; average depth: average sequencing depth, the total number of bases aligned to the reference genome divided by the genome size; coverage at least 1X: percentage of sites covered by at least one base in the reference genome; coverage at least 4X: the reference genome has at least four bases covering the percentage of sites in the genome.

**Table 3:** SNP detection statistics and annotation results.

Category		Number of SNPs
Upstream		40,870
Exonic	Stop gain	244
	Stop loss	36
	Synonymous	35,791
	Non-synonymous	23,807
Intronic		2,976,338
Splicing		120
Downstream		47,182
Upstream/Downstream		1030
Intergenic		5,455,335
Total		8,641,229

Note: Total: the total number of SNPs; Upstream: 1 kb region upstream of the gene; Exonic: the variant is located in the exon region; Stop gain: Mutation of the stop codon is obtained in the gene; Stop loss: a mutation that causes a gene to lose a stop codon; Synonymous: synonymous variation; Non-synonymous: non-tautological variation; intronic: the variation is located in the intronic region; Splicing: the variant is located at the splice site (2 bp in the intron close to the exon/intron boundary); Downstrea: 1 kb region downstream of the gene; Upstream/Downstream: the 1 kb region upstream of the gene is also 1 kb downstream of another gene; Intergenic: the variant is located in the intergenic region; Total: The sum of the components.

**Table 4:** Polymorphisms of candidate genes and population genetic analysis.

Sites	Gene Frequency		Genotype Frequency			Chi-Square Value (χ2)	Polymorphic Information Content	Homozygosity	Heterozygosity	Number of Effective Alleles
1	0.29 (C)	0.71 (T)	0.06 (CC)	0.46 (TC)	0.48 (TT)	2.73	0.33	0.59	0.41	1.71
2	0.56 (G)	0.44 (A)	0.09 (AA)	0.21 (GG)	0.70 (AG)	16.49	0.37	0.51	0.49	1.97
3	0.04 (T)	0.96 (C)	0.94 (CC)	0.01 (TT)	0.05 (CT)	2.52	0.07	0.93	0.07	1.08
4	0.95 (C)	0.05 (T)	0.91 (CC)	0.01 (TT)	0.08 (CT)	4.68	0.09	0.9	0.1	1.11

Note: Rank 1 Representative NC_030818.1 (g. 53666634 T > C); Rank 2 Representative *SHBG* (g. 27088465 A > G); Rank 3 Representative *CAPN12* (g. 49371337 C > T); Rank 4 Representative *FRMD5* (g. 55009283 C > T).

**Table 5:** NC_030818.1 53666634T>C) mutation site association analysis with leg length.

Genotype	Frequency	Leg Length/(cm)
CC	16	24.156 ± 1.080 ^a^
TC	114	28.278 ± 0.404 ^b^
TT	119	30.408 ± 0.396 ^c^

Note: Different letters of the shoulder mark of the same column of data indicate significant differences (*p* < 0.05).

**Table 6:** Primer sequences.

Sites	Chromosome	Gene ID	Upstream Primers (5′–3′)	Downstream Primers (5′–3′)
g. 53666634 T > C	NC_030818.1	102191807	TCCCTCCCCCAAATGTGATG	TCATCTTGTGGGAGCCGATT
g. 40040094 G > C	NC_030826.1	102176826	CCATTGCTGTTGGGCTGGT	AGGCCCTGAACAGTTTACGC
g. 76236896 C > T	NC_030817.1	102179855	AGTGCCATTAGGACCAGCAAG	ATACGGACCTGGTTGGGGTTA
g. 43291383 G > C	NC_030826.1	102185686	GGGATGATTTCCGTGGCATC	TGGCACTATGCAGCTCTCTC
g. 49514296 C > T	NC_030825.1	102181421	TCGTAGTGGCTGGTAAACACA	GAATCACTGCTGCCCAAGGT
g. 27088465 A > G	NC_030826.1	102191733	GCCCACAGCAAGCAAATGAC	CCCTGGCTCAAAACCACCAT
g. 49371337 C > T	NC_030825.1	102178102	GTCCTTGTTCCCGTGAGTGT	GCCCAGCTCATCTGCATCTT
g. 55009283 C > T	NC_030828.1	102186636	ACCACAGGCTTTTCTGGAGG	GAGAGTCAGAGACAAGCGGG

**Table 7:** PCR reaction system.

Component	Volume
Two × SuperTaq PCR StarMix	25 μL
Forward primers (10 μM)	2.5 μL
Reverse primers (10 μM)	5.5 μL
DNA	50 ng
Sterile, enzyme-free water	Complement to 50 μL

**Table 8:** PCR reaction procedure.

Process	Temperature	Time	Number of Cycles
Pre-denaturation	95 °C	2 min	/
Denaturation	95 °C	15 s	35 circulate
Anneal	50–72 °C	15 s	
Extend	72 °C	15 s/kb	
Terminal extension	72 °C	5 min	/

## Data Availability

The original contributions presented in the study are included in the article/Supplementary Materials Figure S1. Further inquiries can be directed to the corresponding authors.

## Supplementary Materials

The following supporting information can be downloaded at: https://www.mdpi.com/article/10.3390/ijms252212450/s1.

## Appendix A

**Table A1:** Body size information of 30 candidate Leizhou goats.

Sample	Body Height/cm	Chest Depth/cm	Leg Lenth/cm
TL1	62.0	34.0	30.5
TL2	63.0	33.5	31.0
TL3	65.0	32.0	32.0
TL4	58.5	34.5	29.0
TL5	60.5	31.5	30.0
TL6	63.5	34.0	31.5
TL7	61.8	30.5	30.8
TL8	62.8	33.5	31.3
TL9	60.0	33.5	30.0
TL10	61.5	33.8	30.8
TL11	58.5	33.3	29.5
TL12	62.0	32.0	31.5
TL13	59.0	31.8	30.5
TL14	58.0	30.3	30.5
TL15	63.0	28.5	33.5
SL1	55.5	31.5	21.5
SL2	57.0	32.0	23.5
SL3	54.5	33.0	22.5
SL4	59.0	29.5	24.5
SL5	54.0	30.5	22.5
SL6	59.5	32.0	25.5
SL7	53.5	31.0	23.0
SL8	59.0	31.5	25.5
SL9	59.0	30.0	25.5
SL10	60.0	30.8	26.3
SL11	59.3	29.0	26.0
SL12	57.5	30.5	25.5
SL13	57.5	28.5	25.8
SL14	55.0	27.5	24.8
SL15	52.0	29.5	23.5

**Table A2:** Candidate SNP locus gene information.

Gene	Chromosome Number	Chromosomal Location	Reference Genomic Bases	Mutant Base
NC_030818.1	NC_030808.1	2469037	A	G
*NR1D1*	NC_030826.1	40040094	G	C
*PARP2*	NC_030817.1	76236896	C	T
*SOST*	NC_030826.1	43291383	G	C
*NFKBIB*	NC_030825.1	49514296	C	T
*SHBG*	NC_030826.1	27088465	A	G
*CAPN12*	NC_030825.1	49371761	C	T
*FRMD5*	NC_030828.1	55009283	C	T
