# Supplementary material for: Screening of SNP Loci Related to Leg Length Trait in Leizhou Goats Based on Whole-Genome Resequencing
Source: Int J Mol Sci. 2024 Nov 20;25(22):12450. doi: 10.3390/ijms252212450 (PMC11594888; doi:10.3390/ijms252212450)
Supplement: Supplementary file 1 [file ijms-25-12450-s001.zip › Figure S1.docx]

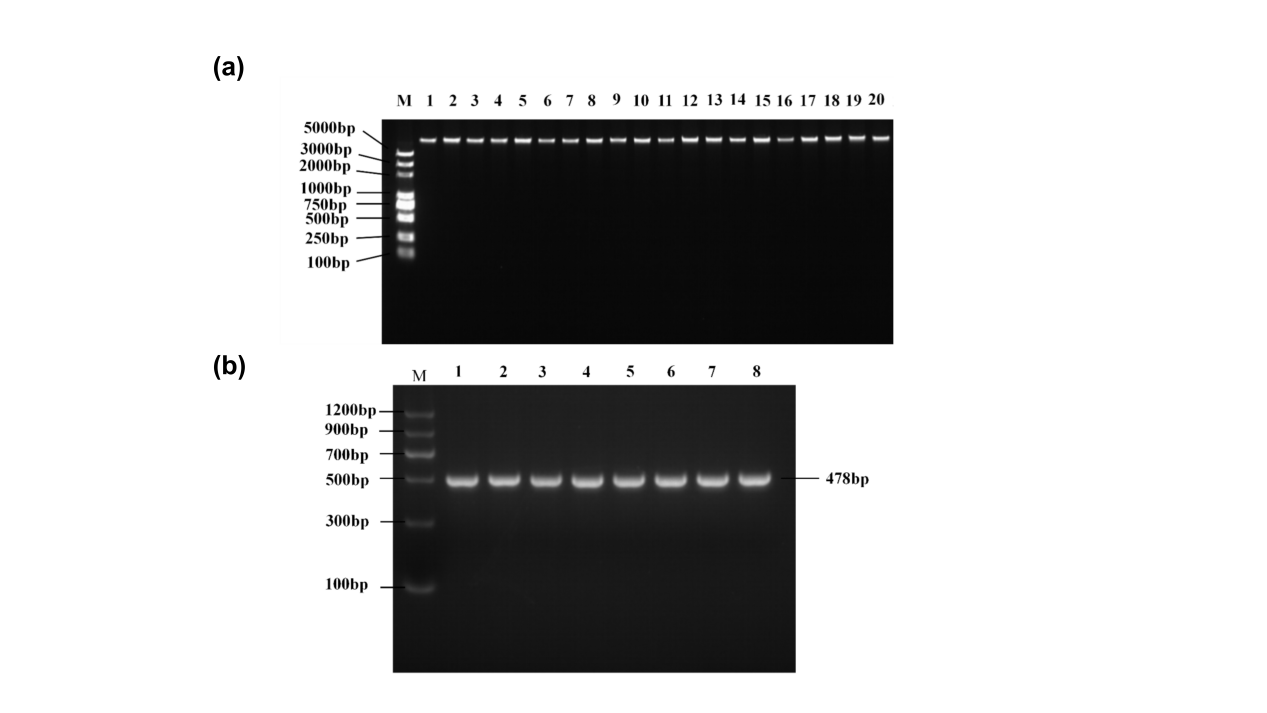


**Figure 7**. (a) Agarose gel electrophoresis of Leizhou goat blood DNA;

(b) PCR product of the fragment located in NC_030818.1 (g. 53666634T> C).
