# Supplementary figures and images for: Screening of SNP Loci Related to Leg Length Trait in Leizhou Goats Based on Whole-Genome Resequencing
Source: Int J Mol Sci. 2024 Nov 20;25(22):12450. doi: 10.3390/ijms252212450 (PMC11594888; doi:10.3390/ijms252212450)

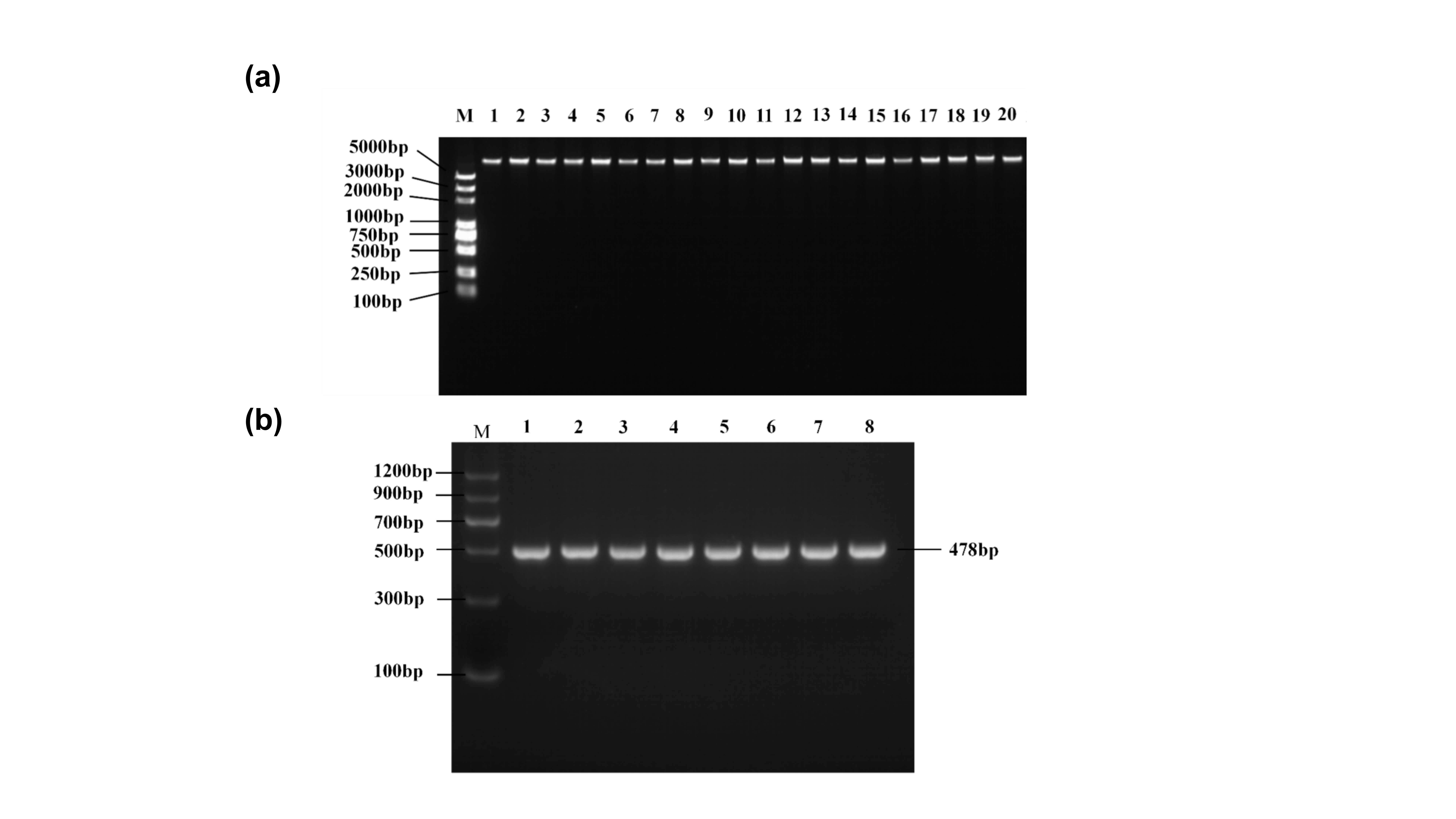

Supplement: Supplementary file 1 [file ijms-25-12450-s001.zip › Figure S1.png]
